# Per- and polyfluoroalkyl substances (PFAS): immunotoxicity at the primary sites of exposure

**DOI:** 10.1080/10408444.2025.2501420

**Published:** 2025-05-22

**Authors:** Emma Arnesdotter, Charlotte B. A. Stoffels, Wiebke Alker, Arno C. Gutleb, Tommaso Serchi

**Affiliations:** a Environmental Sustainability Assessment and Circularity (SUSTAIN) Unit, Luxembourg Institute of Science and Technology, Esch-sur-Alzette, Luxembourg; b Department of Food Safety, German Federal Institute for Risk Assessment, Berlin, Germany

**Keywords:** PFAS, per- and polyfluoroalkyl substances, immunotoxicity, in vitro, lung, intestine, skin

## Abstract

Per- and polyfluoroalkyl substances (PFAS) are persistent synthetic chemicals widely used in industrial and consumer products, leading to environmental contamination and human exposure. This review focuses on perfluoroalkyl acids, a subset of PFAS, which are primarily encountered through diet, including drinking water, and other pathways such as dust ingestion, and dermal contact. Impaired vaccine antibody response has been identified as the most critical effect for risk assessment by the European Food Safety Authority. Furthermore, human epidemiological studies have linked exposure to certain PFAS to various immune-related outcomes, such as asthma, allergies, and inflammatory bowel disease. This review examines potential immunomodulatory effects of perfluoroalkyl acids at the primary sites of exposure: lungs, intestines, and skin, using human epidemiological data as the basis for investigating these impacts. While animal studies are referenced for context, this paper highlights the need for further human-based research to address key questions about PFAS and their immunological impacts. The state of *in vitro* toxicity testing related to these effects is thoroughly reviewed and critical issues pertaining to this topic are discussed.

## Introduction

### What are PFAS and why are they used?

Per- and polyfluoroalkyl substances (PFAS) are a broad chemical class of persistent synthetic compounds used in multiple industrial and consumer applications, including firefighting foam, food packaging, non-stick repellents, and waterproof products. Perfluoroalkyl substances are compounds for which all hydrogen on all backbone carbon atoms (thus excluding the carbon of any functional groups, e.g. the carboxylic group) have been replaced by fluorine, whereas polyfluoroalkyl substances are compounds for which all hydrogen on at least one (but not all) carbon atoms have been replaced by fluorine. Hence, the Organisation for Economic Cooperation and Development (OECD) Global Perfluorinated Chemicals Group defines PFAS as “*fluorinated substances that contain at least one fully fluorinated methyl or methylene carbon atom”*. This means that, with a few exceptions, any chemical with at least one perfluorinated methyl group (–CF_3_) or a perfluorinated methylene group (–CF_2–_) is a PFAS (OECD 2021).

**Figure 1.**
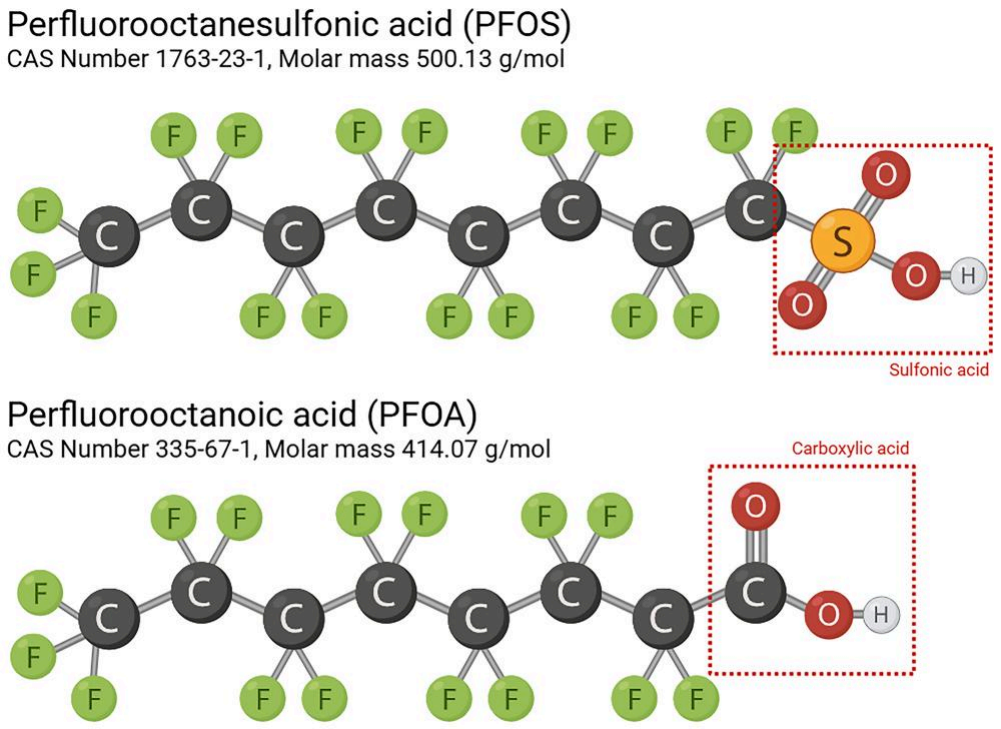
The conventional PFAS nomenclature is based on the total number of carbon atoms in the molecule. Note that the functional group may contain one or more carbon atoms, which are included in the total number of carbons in the compound name. This is illustrated in the figure with PFOS and PFOA. Created in BioRender. Arnesdotter, E. (2025) https://BioRender.com/y16i059.

Perfluoroalkyl acids (PFAA) represent a widely used subclass of PFAS, characterised by a fully fluorinated carbon chain and a functional group. These functional groups are typically either a sulphonate (perfluorosulfonic acids, (PFSA)) or a carboxylate (perfluorocarboxylic acids, (PFCA)), which serve as the basis for subclassification (Buck et al. 2011). PFAA are further distinguished by chain length, with the OECD defining long-chain PFAA as PFCA containing ≥7 and PFSA containing ≥6 carbon atoms (OECD 2021) though, definitions may vary across jurisdiction. PFAS nomenclature conventionally reflects the total number of carbon atoms in the molecule (Figure 1).

**Figure 2.**
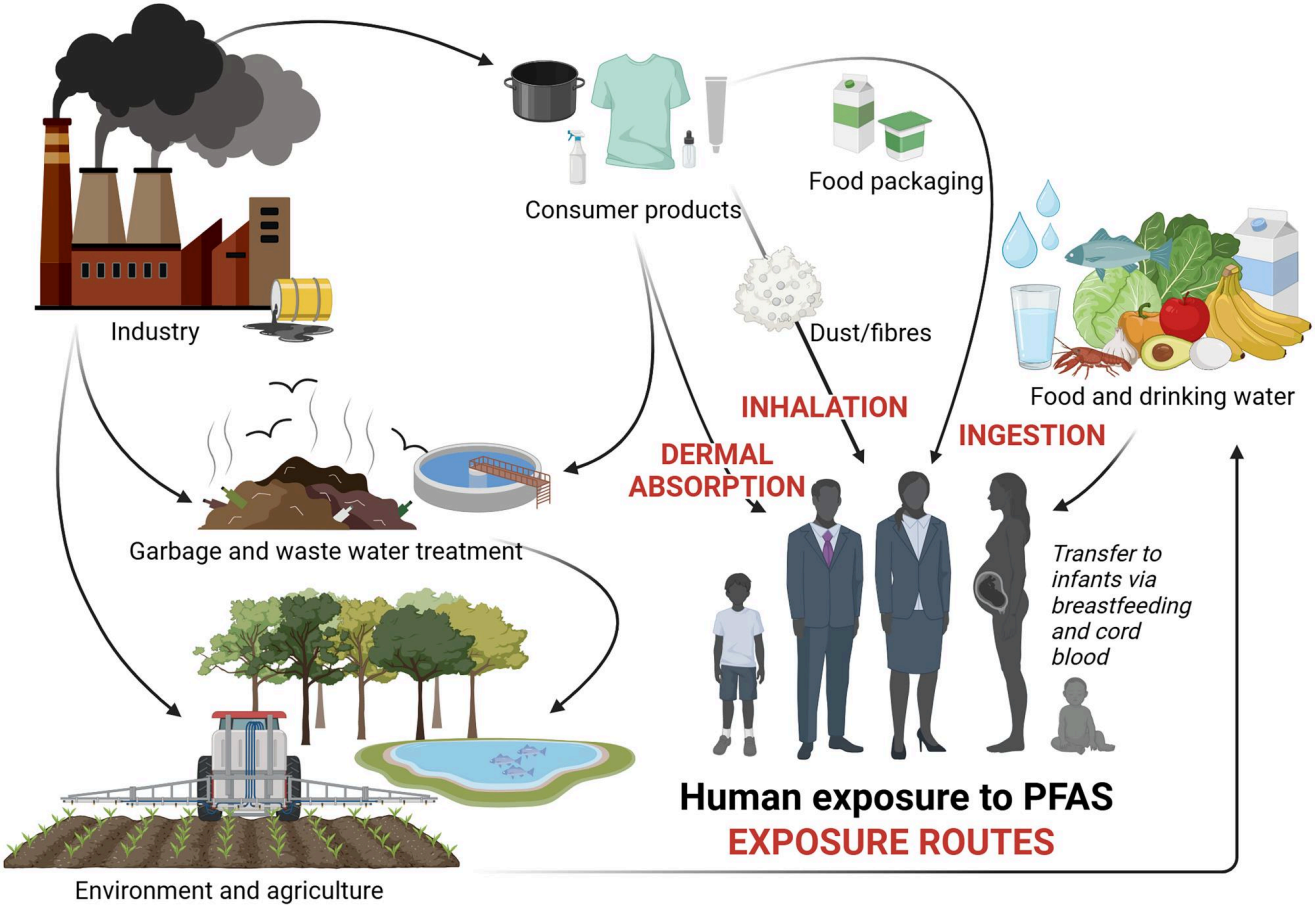
Human exposure to PFAS and relevant exposure routes. As a result of their widespread use and environmental persistence, human exposure to PFAS has become a significant concern for public health. Three primary routes of exposure have been identified for PFAS: inhalation, ingestion, and dermal contact. Each route presents distinct pathways by which individuals may encounter PFAS in the environment, consumer products, or occupational settings. Adapted from (Sunderland et al. 2019). Created in BioRender. Arnesdotter, E. (2025) https://BioRender.com/v63n894.

PFAS compounds have been produced since the 1940s for their ability to interact between two immiscible fluid phases acting as a surfactant, and their repellent and extremely stable and non-reactive properties (Kissa 2001). PFAS are in general amphiphilic molecules, meaning that they have a hydrophilic part, as well as a hydrophobic and lipophilic part, allowing them to segregate from both polar and non-polar solvents to form their own partition (Leung et al. 2023). The carbon-fluorine bond, among the strongest in organic chemistry, provides PFAS with biological, chemical, and thermal stability, making most of them inert with very limited reactivity (O’Hagan 2008). Regrettably, the desired properties of these compounds also make them very persistent in the environment. Measured levels of legacy PFAS have generally declined since the introduction of regulatory bans and restrictions. However, due to their persistence and mobility, it remains uncertain whether these reductions reflect an overall environmental decrease or a redistribution to compartments or regions that are less frequently monitored. Today, there are more than 4700 different PFAS described in the OECD database that are known, or likely, to have been on the global market (OECD 2018). Thus, it is only a fraction of the thousands of PFAS compounds that are monitored, and it is not possible to conclude if a decrease in one type of PFAS is outweighed by an increase in others. PFAS are used across nearly all industrial sectors and in numerous consumer products. More than 1400 individual PFAS have been identified across over 200 use categories and subcategories, demonstrating their widespread presence in products and processes with direct environmental and human contact, such as food packaging and textiles (Glüge et al. 2020). Due to their stability, PFAS are omnipresent and recalcitrant in the environment, and can be bioaccumulative in plants, and in humans and animals (Ghisi et al. 2019; Wang et al. 2020; Evich et al. 2022). It is noted that individual PFAS compounds have different accumulation potential. PFAA are the most persistent fraction of all PFAS and are regarded as a final degradation product (Brendel et al. 2018). Multiple precursor substances (e.g. from landfill waste) may degrade to PFAA in the environment (e.g. soil, water, and wastewater treatment systems). This is due to microbial processes that partially break down the polyfluorinated precursors, into simpler forms, notably PFAA. This way, PFAA are released into the environment, subsequently leading to human exposure (Figure 2) (Liu and Mejia Avendaño 2013; Allred et al. 2015; Brendel et al. 2018; Hamid et al. 2018). Most PFAS fulfil the ‘very persistent’’ criteria under the European Union (EU)’s Registration, Evaluation, Authorisation and Restriction of Chemicals (REACH) Regulation (EC 2006) and are among organic chemicals seen as the environmentally most persistent substances. This characteristic form the basis for the often-used moniker of ‘forever chemicals’ for PFAS in the popular press, as well as the argument to manage PFAS as a chemical class (Cousins et al. 2020). Large efforts are undertaken to develop remediation and destruction methods. However, these techniques go hand in hand with great efforts, high costs and energy consumption, as well as a limited magnitude compared to the extent of PFAS pollution (Wanninayake 2021; Das and Ronen 2022; Meegoda et al. 2022; Lu et al. 2023).

### The routes of human PFAS exposure

Diet, including drinking water, is the major source of PFAS exposure for most of the population (Figure 2). When PFAS chemicals leach into water sources from industrial sites, waste sites, or areas where firefighting foams have been used, they can contaminate drinking water supplies which lead to human ingestion and exposure in and through the intestinal system. Many PFAS may enter the food chain after being transferred from soil to plants, being particularly represented in milk, eggs, and meat (EFSA CONTAM Panel, 2020; Xu et al., 2020). Moreover, the widespread use of PFAS in various consumer products (*i.e.* personal care products including cosmetics, water-repellent clothing etc.) makes dermal absorption through direct contact with these products a possible source of exposure (EFSA CONTAM Panel, 2020; Kotthoff et al., 2015; Bečanová et al., 2016; Schultes et al., 2018; Whitehead et al., 2021; van der Veen et al., 2020; Harris et al., 2022; Ragnarsdóttir et al., 2022). In cosmetics, PFAS act as conditioners, film forming, solvents and surfactants (KEMI, 2021). Other contributions to human PFAS exposure include dust ingestion and indoor air inhalation (Sunderland et al., 2019).

### Fate and pharmacokinetics of PFAS

The hydrophobicity and lipophilicity of PFAS facilitates them to affect and accumulate in different organs and biological fluids, with plasma, liver, and kidney identified as key sites of bioaccumulation (Maestri et al. 2006; EFSA CONTAM Panel 2020). Many PFAS have been shown to bind to albumin. The binding occurs *via* the fatty acid binding sites, which is probably due to the structural similarities of many PFAS, especially PFCA, with fatty acids, and *via* adsorption to the surface of the protein (Forsthuber et al. 2020; Fedorenko et al. 2021; Jackson et al. 2021; Smeltz et al. 2023). Several characteristics in the molecular structure of PFAS effect the affinity of albumin, such as; the functional group (e.g. higher affinity for sulphonated than carboxylated moieties), the number of fluorinated and aliphatic carbon atoms, the number of fluorine atoms (e.g. for tested PFCA and PFSA highest affinity for compound containing 6–8, 7–9 and 13–17 atoms, respectively) (Jackson et al. 2021), the hydrophobicity (e.g. greater hydrophobicity leads to a higher affinity) (Fedorenko et al. 2021), and whether the structure is linear or branched (e.g. higher affinity for a linear PFAS than its branched isomer) (Beesoon and Martin 2015). Consequently, albumin acts as a transport vehicle, enabling systemic circulation and delivery of PFAS to blood-rich tissues. Indeed, several PFAS have been detected in embryonic and foetal tissues, leading to the conclusion that they may pass the foetus-blood barrier (Mamsen et al. 2019; Sunderland et al. 2019; EFSA CONTAM Panel 2020).

PFAS elimination in humans occur *via* urine and, to a lesser extent, *via* faeces (Han et al. 2012). It has been shown that some organic anion-transporting polypeptides (OATP) can transport certain PFAS. They are expressed in human hepatocytes and/or enterocytes and are therefore believed to facilitate enterohepatic circulation of PFAS (Zhao et al. 2016). The renal elimination involves a passive process *via* glomerular filtration and active pathways of tubular secretion and reabsorption (i.e. the absorption of molecules, ions, and water from the glomerular filtrate back into the blood) (Han et al. 2012). Transporters located at the basolateral or apical membrane of tubular cells (e.g. organic anion transporters (OAT) (Nakagawa et al. 2008; Louisse et al. 2023), OATP (Weaver et al. 2010), and organic solute transporter-alpha/beta (OSTα/β) (Zhao et al. 2015)) mediate the transport of certain PFAS, indicating involvement in renal secretion and reabsorption. Molecular characteristics of PFAS, such as functional groups, molecular size, and carbon-chain length, influence transporter affinities and the proportion of albumin-bound versus unbound PFAS. These factors, in turn, affect both passive diffusion and transporter-mediated processes (Fang et al. 2025), and likely contributes to the differences in half-lives observed among various PFAS. For short-chain PFAS, half-lives range from days to approximately a couple of months (Olsen et al. 2009; Russell et al. 2013; Russell et al. 2015) whereas long-chain PFAS may persist in the body for several years (Olsen et al. 2007; Li et al. 2018), though slightly shorter half-lives have also been reported (Xu et al. 2020).

### Expanding the current knowledge on the toxicity of PFAS

Currently, the most studied PFAA are PFSA (e.g. perfluorooctanesulfonic acid (PFOS)) and PFCA (e.g. perfluorooctanoic acid (PFOA)). Albeit now restricted, PFOS and PFOA have been most heavily used and are therefore often detected in the environment (Wee and Aris 2023) and in biological samples (Göckener et al. 2020). Consequently, there is ample amount of toxicity data on these compounds, which is based mostly on findings from animal studies and on human epidemiological data. Indeed, hazard assessment has been traditionally performed based on data from animal experiments. However, this is time-consuming, costly, and ethically questionable. Moreover, existing toxicokinetic data, in particular for PFOS and PFOA, indicate substantial interspecies differences in toxicokinetic parameters, including compound elimination half-life (Pizzurro et al. 2019). In epidemiological studies, blood concentrations of PFAS (mainly PFOS and PFOA but also other less studied PFAS) are measured in populations worldwide, *e.g.* general population sample in one country at one time point, or specific populations with occupational exposure (Radke et al. 2022). As an alternative to animal testing, *in vitro* studies may be conducted using human-relevant organs, cells, or organelles outside their native context. Given the limited toxicity data for most PFAS congeners and the EU’s commitment to phasing out animal testing, future PFAS hazard assessment should rely on mechanistic approaches known as new approach methodologies (NAMs). NAMs include *in vitro* assays based on human-derived cell models, as well as *in silico* methods such as read-across. NAMs are generally considered faster, cheaper, and potentially more human-relevant (when based on human cell models) than animal studies (Schmeisser et al. 2023).

### Immunomodulatory effects of PFAS

Several human observational studies have investigated potential associations between PFAS (primarily PFOS and PFOA) with immune outcomes, including asthma, allergies, inflammatory bowel disease (IBD), serum antibody response to vaccination, and increased susceptibility to infections. Currently, effects on the immune system, as shown by decreased antibody response to vaccines (Grandjean et al. 2012; Grandjean et al. 2017; Grandjean et al. 2017; Abraham et al. 2020), is considered by EFSA as the most critical effect and is used as the basis of the risk assessment of PFAS exposure through food (EFSA CONTAM Panel 2018; 2020). Similarly, the United States (of America) Environmental Protection Agency (US EPA) considers the suppression of vaccine response in children as the most sensitive (non-cancer) endpoint in human epidemiology studies for PFOS and PFOA (EPA 2022a; 2022b). Consequently, immunomodulatory effects are of great interest to PFAS human health risk assessment.

This review scrutinises possible immunomodulatory effects at the primary sites of exposure (the lungs, the intestine, and the skin) related to the three major routes of exposure (i.e. inhalation, ingestion, and dermal contact), starting from human epidemiological data. This paper does not aim to review the ample animal data available for these compounds, rather such data is used merely in a supportive measure. Further, we aim to provide the state of the science on *in vitro* toxicity testing of such effects, as well as to bring forward critical issues pertaining to this topic.

## Literature review methodology

The latest EFSA report on PFAS (EFSA CONTAM Panel 2020), particularly the human epidemiological data, was consulted to frame the scope of this narrative literature review. The review focuses on immunotoxic effects related to primary sites of human exposure to PFAA, including allergy, asthma, and IBD, as well as potential immunotoxic effects on the skin.

The PFAS-Tox Database (Pelch et al. 2021), containing over 1000 studies, was initially used as the primary source for identifying relevant studies. Filters applied included “Metabolic & Digestive System,” “Respiratory System,” “Immune System,” “Sensory System,” and “Systemic/Non-Specific/Other,” alongside filters for human and *in vitro* studies, yielding in total 366 unique studies. However, as the latest update of this database occurred in January 2021, an additional search was conducted using PubMed to identify studies published between 2021 and October 2024. This search also included studies on the two legacy PFAS compounds, PFOS and PFOA, which are excluded from the PFAS-Tox Database, with no restriction on publication year.

The PubMed search combined keywords such as “PFOS,” “PFOA,” “PFAS,” “*in vitro*," “lung OR intestine OR skin,” “immunotoxicity,” and “inflammation.” Inclusion criteria were limited to articles published in English, peer-reviewed journals, and reporting primary experimental data. Relevant review articles were also examined to provide broader context. Titles and abstracts were screened for relevance, and selected papers were critically evaluated for methodological rigour.

## Toxicity at the three primary sites of exposure

Three primary routes of exposure have been identified for PFAS, namely inhalation, ingestion, and dermal contact. Each route presents distinct pathways by which individuals may encounter PFAS in the environment, when using consumer products, or in an occupational setting. This section aims to inform on potential immune reactions related to these major exposure routes, thereby providing insights into areas of interest for further testing.

### Possible immunomodulatory effects in the respiratory system

**Figure 3.**
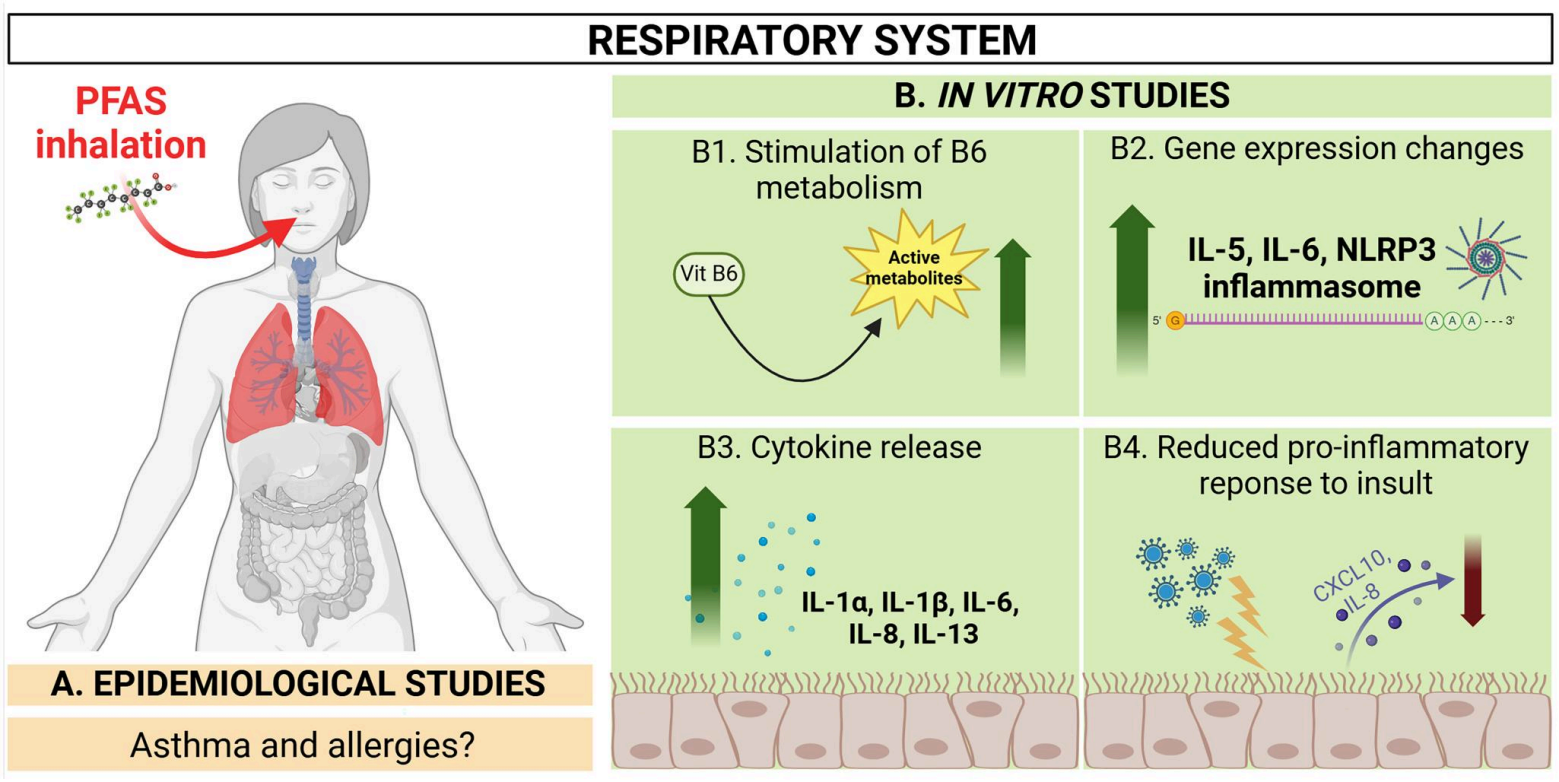
Summary of current *in vitro* findings related to immunomodulatory effects in the lung. Human studies show inconsistent results regarding the relationship between PFAS levels and lung function or asthma prevalence. Some research indicates reduced lung function in children with asthma who are exposed to PFAS, while other studies find no significant connections or produce mixed outcomes. *In vitro* studies have examined the immunomodulatory effects of PFAS, identifying changes in cytokine release, inflammasome activation, and metabolic pathways in lung cells exposed to PFAS. As a result, although the literature offers insights into possible mechanisms linking PFAS exposure to immune toxicity in the lungs, the findings are still inconclusive. Created in BioRender. Arnesdotter, E. (2025) https://BioRender.com/w48z959.

The respiratory system is a potential target for PFAS toxicity through air-borne particles and from the blood circulatory system (Sunderland et al. 2019). PFAS may accumulate in the lung cells through binding to phospholipids (Sanchez Garcia et al. 2018). Suspected effects of PFAS exposure on the lung and current *in vitro* findings are summarised in Figure 3 and described below.

The immune system of the lung consists of a large network of innate immune cells including granulocytes (neutrophils and eosinophils), mast cells, macrophages, dendritic cells, and natural killer cells. Together with their associated inflammatory mediators (i.e. cytokines, chemokines, and complement), they are involved in phagocytosis, neutralisation and killing of infectious agents. The innate immune cells induce adaptive immune responses based on the nature of the pathogen(s). The innate inflammatory cells are also recruited/induced following exposure to chemicals and particles (Greeley 2017; Gopallawa et al. 2023). Like in the rest of the body, the balance of immune responses is key for maintaining immune homeostasis in the lungs. Overreactions may result in diseases such as asthma. PFAS (mainly PFOS and PFOA) have been investigated in relation to asthma and the associated outcomes in numerous rodent studies. A complete review of such studies falls outside the scope of this paper. In short, PFOA has been shown to enhance allergen-induced immunoglobulin E (IgE) production, airway hyperreactivity (Fairley et al. 2007), alter airway function (Ryu et al. 2014), and aggravate T-helper type-2 airway inflammation (Zeng et al. 2021), which could increase the risk and severity of allergy and asthma. However, there are also studies where PFOA and PFOS were not deemed a risk factor for more severe allergic asthma-like symptoms, though PFOA was shown to induce airway inflammation and alter airway function (Ryu et al. 2014). Although some evidence suggests associations between exposure to certain PFAS and asthma, the findings are inconsistent. Moreover, there are known differences in the mode of action (MoA) on how asthma is induced in humans and rodents, which should be kept in mind when assessing animal data on this subject (Pemberton et al. 2024).

#### Associations between human exposure to PFAS and the respiratory system

Studies on the association between PFAS and asthma or allergy-related outcomes in humans are equally inconsistent. In a study of lung function and eight PFAS (PFHxA, PFOA PFNA, PFDA, PFTA, PFBS, PFHxS, PFOS – see Table 1 for full names and CAS numbers) in serum samples of 132 asthmatic children and 168 non-asthmatic controls from a high-exposure area in northern Taiwan, no significant association between these PFAS and pulmonary function was found among children without asthma. However, among children with asthma, three pulmonary function measurements (forced vital capacity (FVC), forced expiratory volume in 1s (FEV1), and forced expiratory flow 25%–75% (FEF25–75%)) was negatively associated with serum levels of certain PFAS, namely PFOS, PFOA, PFHxS and PFNA (Qin et al. 2017). Consistent with these findings, studies involving non-asthmatic adolescents (12–19 years old, *n* = 765) reported no clear associations between serum levels of four PFAS (PFOA, PFOS, PFNA, and PFHxS) and lung function. Nonetheless, when stratifying for age and sex, in 12–15-year-olds, PFNA was negatively associated with FEV1:FVC and FEF25-75% among girls, while PFNA was positively associated with FEV1:FVC among boys (Shi et al. 2023). Prenatal exposure to PFNA has also been positively associated to self-reported asthma, but not doctor-diagnosed asthma or wheeze, in 5-year-old children in a Danish cohort (maternal serum concentration PFNA 0.65 ng/mL). Nevertheless, the findings support immunomodulatory effects of certain PFAS (Beck et al. 2019). Another study examined the association between prenatal exposure (maternal serum) to seven PFAS (PFOA, PFNA, PFDA, PFUnDA, PFHxS, PFHpS, and PFOS) and respiratory health in children up to three years of age but did not demonstrate any adverse effect of prenatal exposure to these PFAS (Philippat et al. 2023). Conversely, a Norwegian study including 675 adolescents showed a strong positive association between serum levels of total PFAS and self-reported doctor-diagnosed asthma (Averina et al. 2019), though no measurements of lung function were performed. In particular, the association was driven by the positive associations between PFOS and PFHxS, and asthma, while other PFAS did not show statistically significant associations when analysed individually. Similarly, PFDA and PFOA were in a US study positively associated with self-reported asthma attacks (in the 12 months prior) in adolescents (Burbank et al. 2023). Regardless, it should be stressed that the latest EFSA report on human health risks related to PFAS presence in food concluded that there are no or inconsistent associations with asthma and allergies for both prenatal and postnatal exposures for PFOS and PFOA (EFSA CONTAM Panel 2020). Similarly, for PFAS other than PFOS and PFOA, the available evidence is deemed insufficient to conclude that exposure can be associated with allergy and asthma in both children and adults. Likewise, a later review of epidemiologic data found only a limited indication of an effect of PFAS exposure on allergic reactions/allergen-specific IgE antibodies, asthma, and lung function (von Holst et al. 2021).

**Table 1.** Individual PFAS discussed in this article.

Full name	Abbreviation	CAS number	Total number of carbons	Functional group
Perfluorobutanoic acid	PFBA	375-22-4	4	Carboxylic acid
Perfluoropentanoic acid	PFPeA	2706-90-3	5	Carboxylic acid
Perfluorohexanoic acid	PFHxA	307-24-4	6	Carboxylic acid
Perfluoroheptanoic acid	PFHpA	375-85-9	7	Carboxylic acid
Perfluorooctanoic acid	PFOA	335-67-1	8	Carboxylic acid
Perfluorononanoic acid	PFNA	375-95-1	9	Carboxylic acid
Perfluorodecanoic acid	PFDA	335-76-2	10	Carboxylic acid
Perfluoroundecanoic acid	PFUnDA	2058-94-8	11	Carboxylic acid
Perfluorotetradecanoic acid	PFTeDA	376-06-7	14	Carboxylic acid
Perfluorobutanesulfonic acid	PFBS	375-73-5	4	Sulphonic acid
Perfluorohexanesulfonic acid	PFHxS	355-46-4	6	Sulphonic acid
Perfluoroheptanesulfonic acid	PFHpS	375-92-8	7	Sulphonic acid
Perfluorooctanesulfonic acid	PFOS	1763-23-1	8	Sulphonic acid
Fluorotelomer alcohol	e.g. 8:2 FTOH	Volatile precursor to PFCA, such as PFOA	8	Alcohol
6:2 Chlorinated polyfluorinated ether sulphonate	Cl-PFESA (F-53B)	73606-19-6	9	Chlorinated polyfluoroalkyl ether sulphonic acid
Hexafluoropropylene oxide (HFPO) dimer acid and its ammonium salt	GenX	62037-80-3	6	Ether Carboxylic acid

Interestingly, a study, published later than the abovementioned EFSA report, distinguished between atopic (triggered by allergens such as pollen or dust) and non-atopic (not related to an allergy trigger) asthma. An association between maternal serum concentrations of both PFOS and PFOA at 24 weeks of pregnancy to non-atopic asthma at age 6 years was demonstrated, albeit no association between childhood concentrations and asthma occurrence was found (Sevelsted et al. 2023). Thus, even though most of the current studies point towards that there is no association between asthma and PFAS, further investigation of non-atopic asthma and PFAS may be of interest. The mechanism of non-atopic (also known as non-allergic and non-type 2 inflammation-mediated) asthma is currently somewhat unclear. Potential causes have been recently summarised in a review (Klain et al. 2022) and include imbalances in neutrophil-driven immune responses resulting from respiratory infections or challenges in resolving inflammation (Uddin et al. 2010; Green et al. 2014). Additionally, activation of an interleukin (IL)-17-dependent pathway may be a contributing factor (Bullens et al. 2006; Raedler et al. 2015; Hudey et al. 2020). To ease readability, the chemokines referenced in this review are not written out in the text but are instead explained in Table 2. In non-atopic asthma, both mucosal and bronchoalveolar lavage samples show increased levels of the chemokine RANTES (also known as Chemokine (C-C motif) ligand (CCL) 5), which is a leukocyte-recruiting proinflammatory chemokine (Appay and Rowland-Jones 2001), and various neutrophil (i.e. a leukocyte and first line of defence)-related mediators such as leukotriene B4, granulocyte-macrophage colony-stimulating factor (GM-CSF), tumour necrosis factor (TNF)α, IL-17A, IL-8, elastase, and metalloproteinase 9 (MMP9) (Peters 2014; Panettieri 2016; Hudey et al. 2020).

**Table 2.** Chemokines mentioned throughout the present review.

Molecule	Function	References
IL-1β	Involved in activation of pro-inflammatory signalling pathways. Signals *via* the same receptor as il-1α.	Lopez-Castejon and Brough (2011)
IL-6	Involved in the proliferation of antibody-producing cells, *i.e.* B -cells and may amplify inflammation.	Choy and Rose-John (2017)
IL-8	Pro-inflammatory cytokine and important mediator of the immune reaction in the innate immune system response with a pivotal role in lung inflammation and disease.	Cesta et al. (2021)
IL-13	Involved in Th2 inflammation, believed to be a possible therapeutic target in the treatment of asthma.	Marone et al. (2019)
IL-5	Involved in the maturation of eosinophils and their release to the circulation.	Greenfeder et al. (2001)
CXCL10	Thought to play an important role in recruiting activated T cells into sites of tissue inflammation.	Dufour et al. (2002)
IL-1α	Pro-inflammatory cytokine. Has a central role in regulating the inflammatory response. Signals *via* the same receptor as IL-1β.	Malik and Kanneganti (2018)
CXCL1	Recruiting and activating neutrophils for microbial killing by activating the release of proteases and reactive oxygen species.	Sawant et al. (2016)
CXCL2	Produced by activated monocytes and macrophages, attracts neutrophils.	Zhou et al. (2023)
CXCL9	Secreted by leukocytes, macrophages, and fibroblasts. Regulates differentiation of naive t cells to t helper 1 (Th1) cells.	Zhou et al. (2023) and Tokunaga et al. (2018)
CCL20	When coupled to it\s receptor, it is responsible for the chemoattraction of immature dendritic cells, effector/memory T-cells and B-cells and plays a role at skin and mucosal surfaces under homeostatic and inflammatory conditions.	Schutyser et al. (2003)
TNFα	Has a central role in the pro-inflammatory response. Promotes inflammation by induction of the expression of inflammatory genes as well as by inducing cell death.	van Loo and Bertrand (2023)
IL-17A	Pro-inflammatory cytokine produced by activated T-cells and various innate immune cell populations in response to IL-1β and IL-23. Induces CXCL8 (IL-8) and recruit neutrophils.	Bullens et al. (2006) and Mills (2023)
IL-18	IL-18 signalling amplifies ongoing inflammatory programmes. *IL18* transcription can be induced by inflammatory stimuli such as lipopolysaccharide (LPS) and interferons.	Landy et al. (2024)

Note: Chemokines are small signalling proteins that play a crucial role in the immune response to injury (e.g. following chemical exposure). Chemokines are released by various cells at the site of exposure or injury whose primary function is to attract immune cells to the affected area. This process, known as chemotaxis, ensures that immune cells can rapidly reach the affected area, initiate inflammation, and promote tissue repair. Chemokines also help regulate the activation and differentiation of these immune cells, shaping the overall immune response to the injury.

#### In vitro data on the immunomodulatory effects in the respiratory system

Despite the many recent advancements in cell culture technologies, replicating complex physiological processes and organ-specific functions of the lung without the use of intact animals is still a challenging task. Thus, many of the effects included in epidemiological and *in vivo* studies (lung function and respiratory volume etc.) are challenging to evaluate in an *in vitro* setting. Still, given the many differences between animal and human lung physiology (Greeley 2017; Pan et al. 2019), it is not only for ethical reasons but also for scientific reasons that animal models are being replaced by *in vitro* models of the human lung for the investigation of adverse effects of chemical exposure. Much progress has been made, and several reviews are providing great overviews of the current possibilities and remaining challenges of *in vitro* modelling of the human lung (Miller and Spence 2017; Nossa et al. 2021). Another review addresses airway remodelling, which constitutes the hallmark of asthma leading to airway hyper-responsiveness and airway obstruction (Zhou et al. 2023). Thus far, the investigation of the immunomodulatory effects of PFAS in *in vitro* models of the lung has mostly focused on the measurement of various chemokines with and without prior stimulation of the cells.

Perturbation of pathways related to the metabolism of vitamin B6, histidine (the precursor for histamine)- and arginine biosynthesis has been observed in lung epithelial A549 cells following PFOA exposure (300 μM, non-cytotoxic concentration, herein defined as >90% cell viability at the tested concentration) (Zhang et al. 2020; 2021) (Figure 3 B1). The metabolism of vitamin B6 was stimulated and levels of vitamin B6 metabolites were increased after PFOA exposure. This is interesting because pyridoxal phosphate, the active form of vitamin B6, is known to alleviate asthma symptoms (lung inflammation and eosinophil density) when supplemented to asthmatic patients, suggesting that vitamin B6 concentration may be directly controlling immune responses that lead to the development of allergic airway disease (Turnquist 2023). In contrast, histamine and other metabolites associated with histidine metabolism increased in PFOA-treated cells (Zhang et al. 2021). Histamine is a known mediator of allergic inflammation through smooth muscle contraction and increased airway permeability (Yamauchi and Ogasawara 2019). Additionally, the levels of IL-1β, IL-6, IL-8, and IL-13 were about 1.5 times higher in PFOA-treated A549 cells compared to the controls (Figure 3 B3). Polyinosinic-polycytidylic acid (Poly I:C) stimulated (mimicking a viral infection) cells showed aggravated levels of IL-1β and IL-6 following PFOA exposure. When vitamin B6 was supplemented into the cell culture the levels of IL-6 and IL-8 were decreased (Zhang et al. 2021).

In human bronchial epithelial BEAS2B cells, exposure for 24h to a non-cytotoxic concentration (defined by the authors based on a cell viability assay) of 300 µM PFOA caused an increase in mRNA levels of IL-5 and IL-6. Exposure for 24h to a non-cytotoxic concentration of 275 µM PFOS increased the mRNA levels of IL-6 and NOD-like receptor protein 3 (NLRP3) inflammasome (Dragon et al. 2023) (Figure 3B2). NLRP3 is an intracellular sensor of endogenous danger signals (Swanson et al. 2019) and a regulator of the innate immune system and inflammatory signalling (Blevins et al. 2022), which in recent years has been linked to the activation and the development of airway inflammatory diseases (Leszczyńska et al. 2022). The formation and activation of the NLRP3 inflammasome lead to a caspase-1-dependent release of the pro-inflammatory cytokines IL-1β and IL-18, and to gasdermin D-mediated pyroptotic cell death (Swanson et al. 2019). The latter is a form of cell death triggered by pro-inflammatory signals and associated with inflammation (Yu et al. 2021; Dai et al. 2023).

Immunomodulatory effects have also been investigated in human bronchial epithelial HBEC3-KT cells exposed to PFAS: three sulphonic (PFBS, PFHxS and PFOS); one carboxylic (PFOA); and a fluorotelomer alcohol (8:2 FTOH), which are volatile precursors of PFCA. The compounds were tested at non-cytotoxic concentrations (≤10 µM, defined by the authors based on a cell viability assay), with and without priming with Poly I:C. None of the tested PFAS induced any significant changes in cytokine release at the tested concentrations in non-stimulated cells. However, a concentration-dependent reduction of Poly I:C-induced secretion of IL-8 and interferon gamma-induced protein (CXCL) 10 was observed in PFOS-exposed cells (Figure 3B4). An increase in IL-1α release was also observed. Moreover, PFOS and PFOA enhanced the release of IL-1β in response to stimulation with Poly I:C whereas PFBS, PFHxS and 8:2 FTOH did not induce any statistically significant changes in cytokine release (Sørli et al. 2020). It should be noted that Zhang et al. (2021) used a 30× higher concentration (i.e. 300 µM) of PFOA compared to Sørli et al. (2020). This, in addition to the use of different cell lines, likely contributed to the discrepancy in the response. Nevertheless, taken together this may suggest that exposure to certain PFAS can influence the inflammatory response in the lung.

**Figure 4.**
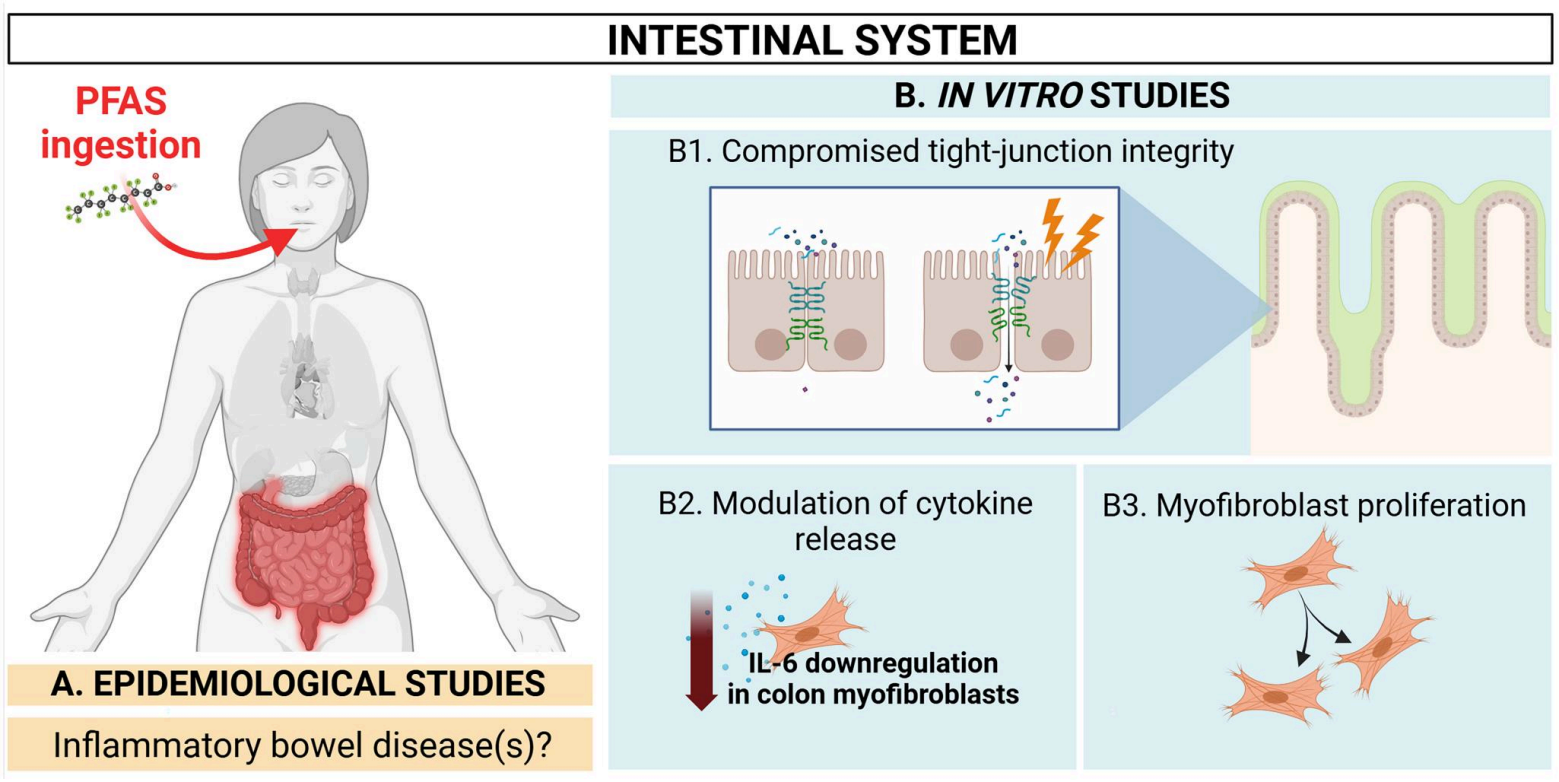
Summary of current *in vitro* findings related to immunomodulatory effects in the intestines. Elevated serum levels of specific PFAS have been associated with late-onset ulcerative colitis (UC), though the evidence is inconsistent, as some studies do not establish a clear link between PFAS exposure and the risk of inflammatory bowel disease (IBD). PFAS might impact the integrity of tight junctions in the intestinal epithelium, potentially increasing permeability and enabling environmental antigens to trigger immune responses linked to intestinal autoimmune disorders. *In vitro* studies show that PFAS can compromise tight junction integrity and alter inflammatory responses in intestinal cells. However, the results are mixed, with some studies even suggesting a suppression of inflammation, which contrasts with expectations for IBD. Created in BioRender. Arnesdotter, E. (2025) https://BioRender.com/h25s692.

#### Summary of the effects in the respiratory system

There are several mechanisms related to immune toxicity outcomes in the lung that potentially could be induced in response to PFAS exposure. Currently, mixed findings on PFAS exposure and asthma/allergy outcomes exist in both animal- and human studies. While some animal research shows adverse effects, human studies indicate inconsistent associations between levels of the tested PFAS and lung function or asthma prevalence. The available data provide insufficient evidence to adjudge the associations between exposure to the tested PFAS and asthma, in particular for the case of non-atopic asthma in offspring. *In vitro,* alterations in cytokine release, inflammasome activation, and metabolic pathways have been seen in cells exposed to different PFAS. However, the available *in vitro* data of PFAS appear rather scattered and the advantages of using *in vitro* methods, such as the possibility of an in-depth investigation of complex mechanisms, are currently not being fully exploited.

### Possible immunomodulatory effects in the intestinal system

Intestinal absorption of most PFAS is known to occur to a significant extent in mammals (Lupton et al. 2012; Juhasz et al. 2025), including humans (EFSA CONTAM Panel 2020). *In vitro,* uptake of PFCA has been demonstrated in human colorectal adenocarcinoma Caco-2 cells to involve a Na^+^-independent and pH-dependent (increased uptake at lower pH), saturable process through OATPs as well as through passive diffusion at concentrations higher than 20 µM (Kimura et al. 2017; 2020). Suspected effects of PFAS exposure on the intestine and observed effects *in vitro* are summarised in Figure 4 and described below.

The gastrointestinal (GI) tract, including the oesophagus, stomach, and intestine, is a complex barrier that acts as the first line of defence against the invasion of ingested microorganisms and toxins. Its’ correct functioning is pivotal to protecting the intact organism while simultaneously allowing the absorption of essential fluids and nutrients in the small intestine (Bischoff et al. 2014). The small intestine is highly folded and comprises several cell types, the major being enterocytes, goblet cells, Paneth cells and stem cells, connected by tight-junction complexes (Fedi et al. 2021). The cell layer is reinforced by mucus, which serves as a defence mechanism against bacterial invasion and plays a critical role in maintaining gut homeostasis. Altered mucus production (due to for example host’s inflammatory state and microbiota composition) can affect the host’s susceptibility to infection (Di Tommaso et al. 2021; Wiertsema et al. 2021). A compromised gut barrier due to, for example alterations in thickness or composition of the intestinal mucus layer, defects in the process of autophagy, and unresolved endoplasmic reticulum stress are, among other mechanisms, thought to play a crucial role in the pathogenesis of the IBDs Crohn’s disease (CD) and ulcerative colitis (UC) (Antoni et al. 2014).

The large intestine, also known as the colon, acts as the reservoir for the community of microbes, including bacteria, viruses, and fungi, that inhabit the GI tract, which are collectively referred to as the gut microbiota. Through co-evolution, bacteria flourish in this habitat while also regulating the host’s physiological functions, including immunity against pathogens, and assisting with the breakdown of food (Lazar et al. 2018). These bacteria are called *commensal bacteria*. The composition of the gut microbiota is shaped by factors like genetics, nutrition, stress, and environmental elements. Changes in microbiota due to exposure to xenobiotics, including environmental pollutants, can lead to inflammation, reduced resistance to pathogens, and altered infection susceptibility (Natividad and Verdu 2013). Moreover, pollutant-induced alterations of gut microbiota are believed to contribute to pollutants’ toxicity (Claus et al. 2016).

One of the key characteristics of amphiphilic compounds such as PFAS is their surfactant properties. Surfactants are a diverse group of compounds capable of adsorbing at the boundary between different phases where they effectively reduce the energy required to break through the surface tension (Kancharla et al. 2022). Surfactants are known to cause dysfunction in tight junctions, which have a central role in regulating the permeability of the intestinal epithelium (Mine and Zhang 2003; Lechuga et al. 2023). Surfactants are even used as intestinal permeation enhancers, which is a widely tested strategy to improve oral delivery of certain drugs (Maher et al. 2016). It is therefore not far-fetched to assume that an increased ingestion of surfactants may have an impact on the permeability of the GI-tract. Dysfunction of tight junctions is a common attribute of intestinal autoimmune diseases such as IBD. When the barrier is compromised, an immune response to environmental antigens that traversed the gut mucosa may develop, leading to intestinal autoimmune diseases, such as Coeliac disease or food allergies (Visser et al. 2009). However, the aetiology of IBD is not yet fully elucidated. The prevailing theory relies on an exaggerated response of the intestinal immune system to the gut microbiota, likely influenced by poorly understood environmental factors as well as a significant genetic component (Loddo and Romano 2015).

PFOA has been shown to disturb both the microbiota composition and the metabolic profiles of the faeces, serum, and liver in male C57BL/6J mice exposed for four weeks to 1 ppm PFOA in drinking water (Gao et al. 2023). Moreover, in female CD1 mice dosed through oral gavage with 1, 5, 10, and 20 mg/kg/day for 10 days, PFOA triggered DNA methylation changes and induced changes in the expression of genes essential for maintaining the gut barrier (*CLDN*, *OCLN*, and *TJP*) with greater effects observed in the small intestine compared to the colon (Rashid et al. 2020). Detrimental effects on intestinal permeability on the small and large intestine have also been shown in *ex vivo* murine tissues following 100 and 10 µM PFOA exposure, respectively (Fart et al. 2021). Also the PFOS alternative F-53B has been shown to cause gut barrier dysfunction and colonic inflammation after sub-chronic exposure in both female and male mice (Pan et al. 2019).

#### Associations between human exposure to PFAS and the gastrointestinal tract

Several studies have investigated possible associations between PFAS and intestinal barrier disruption-related diseases in humans. According to the National Academies of Sciences, Engineering, and Medicine (NASEM) there is suggestive evidence of an association between PFAS and UC in human adults (National Academies of Sciences et al. 2022). UC is an autoimmune relapsing inflammatory disease of the colon that results in diffuse friability and superficial erosions of the colon (Ungaro et al. 2017). Increased total PFAS serum levels (compared to control subjects) have been found in Swedish patients diagnosed with late-onset (≥55 years) UC, but not in patients with CD (Fart et al. 2021). An association between UC and serum levels of PFOA, but not PFOS, PFHxS and PFNA, has been observed also in younger US populations (Steenland et al. 2013; Steenland et al. 2015; Steenland et al. 2018). In both the Swedish and the US studies no association to CD was identified. In contrast, other studies have found no consistent evidence to support PFAS exposure as a risk factor for IBD in a highly exposed Swedish population (Xu et al. 2020), as well as in a US cohort of a population with higher PFAS serum levels compared to the Steenland studies (Lochhead et al. 2022). The Swedish study also included faecal measurements of calprotectin, which is a neutrophil cytosolic protein and a mediator of chronic inflammation, and zonulin, which is believed to be reflective of intestinal permeability. No evidence of a positive association between PFAS exposure and the subclinical risk of IBD was identified. Interestingly, a trend of decreased calprotectin across increased exposure was found, indicating that lower gut inflammation was associated with higher exposure. The causality was not clear (Xu et al. 2020).

#### In vitro data on the immunomodulatory effects in the gastrointestinal tract

Non-cytotoxic concentrations of PFOS (100 µM, defined by the authors based on a cell viability assay) compromise tight-junction integrity in Caco-2 monolayers (Glynn et al. 2017) (Figure 4B1). If the intestinal permeability is increased, cells that normally lie beneath the epithelial layer, such as fibroblasts, may become exposed to the luminal content, including chemicals. Prolonged exposure (96 h) of human colon myofibroblasts CCD-18Co to low levels (≤1 µM) of PFOS and PFOA induce cell proliferation *in vitro* (Giménez-Bastida et al. 2015) (Figure 4B3), which is an important mechanism in intestinal inflammation (Valatas et al. 2017). Fibroblastic proliferation is associated with fibrosis, which is known to alter organ function of some organs, including the liver and the lungs. The role of fibrosis in the GI-tract is, however, less known (Park et al. 2023). PFOS also downregulated IL-1β-induced (mimicking an inflamed condition, 1 ng/mL) IL-6 production at non-cytotoxic concentrations (6 µM, defined by the authors based on a cell viability assay) when exposed in foetal bovine serum (FBS)-deprived cell culture medium (Giménez-Bastida et al. 2015) (Figure 4B2), indicating a suppression of the inflammatory response. Interestingly, this response is the inverse of what would be expected for IBD and is somewhat in contrast to what was seen in human lung cells such as alveolar A549 cells (Zhang et al. 2021), bronchial epithelial BEAS2B cells (Dragon et al. 2023), and HBEC3-KT cells (Sørli et al. 2020), in which exposure to certain PFAS aggravated the inflammatory response. Several PFAS are known immunosuppressors and thus it is conceivable that they have anti-inflammatory properties. Anti-inflammatory effects of several PFAS have been suggested in relation to hepatotoxic (Bassler et al. 2019) and to neurotoxic effects (Gallo et al. 2013). There is evidence that both PFOA and PFOS exposure suppress the release of pro-inflammatory chemokines such as CXCL2, IL-8, CXCL9, and CXCL10, in several human cell lines (Sørli et al. 2020; Szilagyi et al. 2020). Lastly, a recent systematic analysis of PFAS exposure in all taxa concluded that PFAS may induce both immunosuppression and chronic inflammation (Zhang et al. 2023). This appears somewhat contradictory but is in line with a study using non-targeted metabolomics that demonstrated a difference in PFOA toxicity among human cells derived from different organs. Specifically, in A549 cells, accumulation of histamine was observed (discussed in the lung section above) together with increased levels of pro-inflammatory cytokines (IL-1β, IL-6, IL-8, and IL-13), whereas colorectal adenocarcinoma DLD-1 cells showed purine metabolism disruption and cell cycle arrest (Zhang et al. 2021).

### Immunomodulatory effects in the skin

**Figure 5.**
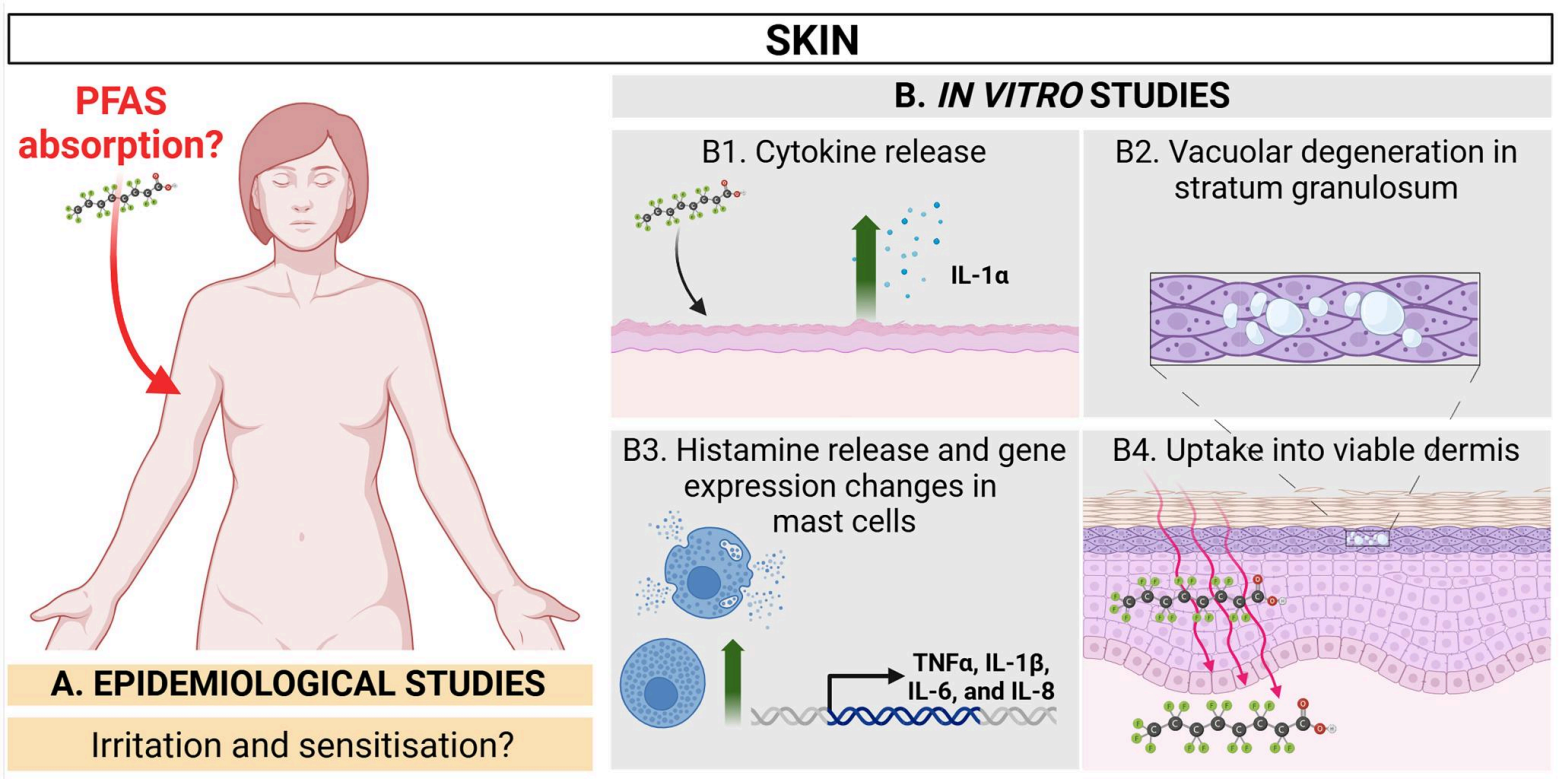
Summary of current *in vitro* findings related to immunomodulatory effects in the skin. The extent to which PFAS are absorbed through the skin into the human bloodstream remains unclear. However, certain PFAS, including some alternatives, have demonstrated the ability to penetrate rodent skin and cause toxic effects. *In vitro* studies, especially those using human skin models, have shown that PFAS can be absorbed and can induce inflammation, though these studies have limitations in accurately replicating real-world exposure scenarios. Created in BioRender. Arnesdotter, E. (2025) https://BioRender.com/e49p784

Immunotoxic effects in the skin include two scenarios with distinct mechanisms of actions: skin irritation and skin sensitisation. Skin irritation is a local and non-allergic inflammatory response to irritants, where the main pathological mechanisms include skin barrier disruption, induction of a cytokine cascade (IL-1α, TNFα, IL-8, and CCL20), and oxidative stress (Fluhr et al. 2008). Skin sensitisation involves an allergic reaction orchestrated by the adaptive immune system to specific allergens. Once an individual has been sensitised, even minimal exposure to the allergen can elicit a robust allergic response (Basketter et al. 2008). However, for this reaction to occur the chemical must penetrate the skin at least as far as the viable epidermis. The human skin is composed of three layers: the subcutis, dermis, and epidermis. The outermost epidermis consists mainly of keratinocytes, which reach a terminal differentiation resulting in the assembly of the stratum corneum. This layer of cornified keratinocytes acts as a physical barrier against many substances (Abdo et al. 2020). In this respect, it should be noted that many PFAS in cosmetics are in ionic or polymeric form, thereby making an extensive uptake unlikely (Putz et al. 2022). Under the stratum corneum are viable keratinocytes, which have a key role in the immune surveillance of the epidermis. Upon stimulation, they trigger the inflammatory response. Together with fibroblasts, endothelial cells, and immune cells such as mast cells, mostly resident in the dermis, keratinocytes play a key role in the initiation, modulation, and regulation of inflammation (Abdo et al. 2020). Suspected effects of PFAS exposure on the skin and current *in vitro* findings are summarised in Figure 5 and described below.

Currently, dermal uptake into the systemic circulation of a wider range of PFAS is not clear (Ragnarsdóttir et al. 2022). Though, recent evidence suggests that the type of emulsion used in topical formulations can significantly influence PFAS bioavailability, with water-in-oil emulsions resulting in greater dermal absorption than oil-in-water emulsions (Chen et al. 2024). The available data further indicate that PFOA can be absorbed through both human and murine skin, but it is strongly dependent upon the ionisation status of PFOA (Franko et al. 2012; Abraham and Monien 2022). A fractional absorption value of 1.6% in human skin under finite dose conditions has been suggested based on a review of dermal absorption kinetics (Yeh et al. 2025). While dermal absorption of PFOA may be relatively low compared to ingestion, it remains a relevant exposure pathway due to the widespread use of these chemicals in consumer products. Several other PFAS, including the so-called “alternatives,” have been shown to penetrate and exert toxic effects on murine skin. For the case of PFCA, daily PFOA exposure for four days increased neutrophils and gene expression of inflammatory cytokines *IL1B* and *IL6* as well as Th2-type skewing cytokine *TSLP*, while 14 days exposure reduced total cell number and decreased the expression of *PPARA*, which is involved in immune cell differentiation and fate commitment (Christofides et al. 2021), and *NFKB1*, which is a key cellular driver of inflammation and immunity (Somma et al. 2021). Expression of genes associated with skin barrier integrity (*FLG2, ITGBL1, LOR*) decreased with prolonged PFOA exposure (Shane et al. 2020). 28-day exposure to PFPeA, PFHxA, and PFHpA led to increased immune cell frequency and dose-related histological changes in the skin including hyperplasia, hyperkeratosis, and inflammation. Gene expression analysis revealed increases in inflammatory cytokines (e.g. *IL6*), Th-2 cytokines (e.g. *TSLP*), and necrosis-related genes (e.g. *SERPINE1*). PFPeA, PFHxA, and PFHpA affected PPAR expression differently. Specifically, *PPARA* decreased with PFPeA and PFHxA exposure, but not with PFHpA. *PPARG* decreased with all tested PFAS. Additionally, alterations were observed in skin barrier integrity genes, with PFHxA and PFHpA increasing *Lor* gene expression and PFPeA and PFHpA increasing *FLG* and *KRT10* expression (Weatherly et al. 2023). Similarly, PFBA caused increases in various immune cell types and alterations in skin structure, including hyperplasia, hyperkeratosis, erosion, fibrosis, and necrosis. These changes were accompanied by increased gene expression of *IL1B*, *IL6*, Th2 cytokine *TSLP*, and *SERPINE1. PPARA* decreased with PFBA exposure. Chemokines *CXCL1* and *CXCL2*, which acts as a chemoattractant for several immune cells, also showed significant increases. In contrast to the other tested PFAS, no changes were observed in skin barrier integrity genes (Weatherly et al. 2021). For the case of 28 days exposure to the sulphonic acid PFHxS (15.63–125 mg/kg/dose), despite that no histological changes were seen, *IL6* increased along with *TSLP, CXCL1, S100A8, SERPINE1*, and *PPARD*, while *PPARG* decreased, indicating immunotoxicity without overt toxicity at the highest concentration tested. In contrast to PFOA, skin barrier genes *LOR* and *FLG* increased (Weatherly et al. 2024). Taken together, these findings underscore the diverse and complex responses of the skin and immune system to different PFAS exposures, suggesting a potential to disturb the dermal barrier and exert toxicity through modulation of cytokines, gene expression, and immune cell populations. Therefore, it remains highly relevant to assess the possibility of human dermal uptake and toxicity of non-ionizable and low-molecular PFAS.

#### In vitro data on the immunomodulatory effects in the skin

To the extent of our knowledge, there are no human epidemiological studies investigating the associations of PFAS exposure and skin irritation or skin sensitisation. However, in recent years there have been an increasing number of *in vitro* studies published. When assessing *in vitro* studies on possible PFAS uptake into cells and subsequent effects, the true physiology of the skin must be kept in mind. In intact skin, a chemical first needs to penetrate through the stratum corneum to reach the viable cells below, which is pivotal to consider in an *in vitro* model of the skin.

Uptake of PFOA (diluted in acetone) has been demonstrated *in vitro* using full-thickness human skin (samples obtained from surgical procedures). Following 24 h, 24% of the applied PFOA had penetrated the skin whereas 45.6 ± 5.2% was found in the skin (Franko et al. 2012) (Figure 5B4). However, the potential impact of using acetone as solvent should be considered, as it may enhance the uptake of certain compounds, including amphipathic substances, by increasing skin permeability (Tsai et al. 2001; Rissmann et al. 2009). The EpiDerm Full Thickness (EpiDermFT) model is a human skin equivalent model for *in vitro* skin evaluation composed of human epidermal keratinocytes and human dermal fibroblasts cultured to form a multilayered and highly differentiated epidermis and dermis. Inflammation was evaluated in a study with the EpiDermFT model exposed every other day over six days to four PFCA, namely PFPeA, PFHxA, PFHpA and PFOA (C5-8) at two concentrations (0.25 and 2.5 mM), using 20 µL per application. Skin histomorphology remained normal in all treatment conditions apart from the high concentration of PFOA where vacuolar degeneration was observed in the stratum granulosum of the epidermis (Figure 5 B2), as well as a decreased thickness compared to control tissues. Yet, this was not sufficient to reduce cell viability by measurement of cell proliferation. After 24 h exposure to 2.5 mM of the four PFAS, pro-inflammatory IL-1α levels were increased in exposed tissues, but these differences were not statistically significant compared to controls, possibly due to a high replicate variation. IL-1α decreased over time in all samples. Only PFOA caused a significant increase in IL-1α levels at the later time points (Figure 5B1), thus indicating a possibility to induce keratinocyte membrane perturbation (Han et al. 2020). It should be noted that the dose used (three times application of 20 µL 2.5 mM PFOA) is several times greater than any plausible consumer exposure scenario.

Mast cells are key effector cells of allergic reactions. They are located in the dermis, near blood vessels, nerves and hair follicles. They contain a large amount of pre-formed pro-inflammatory mediators embedded in secretory granules. This facilitates a rapid response and initiation of further immune effector cell recruitment (Voss et al. 2021). Mast cells bind soluble IgE antibodies produced by B cells after sensitisation to a specific allergen and respond by releasing histamine, which is the main driver of the allergic reaction (Allen 2022). In this respect, PFOA (25–100 μM, 24 h) has been shown to induce histamine release in a time- and concentration-dependent manner in HMC-1 cells. Additionally, PFOA induced gene expression of *TNFA*, *IL1B*, *IL6*, and *IL8* (Singh et al. 2012) (Figure 5B3). However, for this to happen *in vivo*, the compound first needs to cross the stratum corneum. Interestingly, in the animal studies summarised above, the authors observed a decrease in splenic B cell population (Shane et al. 2020; Weatherly et al. 2021; 2023; 2024) which could, at least in theory, result in a lesser mast cell response.

Surfactants (thus possibly PFAS) are known to cause skin irritation by disrupting the keratinocyte membrane, leading to IL-1α release, and further inducing the expression of IL-6 and IL-8, which is followed by morphological changes (Welss et al. 2004). However, not all surfactants necessarily have the same potential to induce irritation. Fluorosurfactants, such as PFAS, lack the lipophilic tail of other hydrocarbon surfactants, which could influence the mechanism of toxicity (Welss et al. 2004). Considering also the IL-1α release in the EpiDermFT discussed above, investigating a potential inflammatory response to PFAS in the skin may be of relevance. Furthermore, like the gut the skin also harbours a microbiome (Byrd et al. 2018), which may be sensitive to environmental pollutants. Thus, if PFAS are demonstrated to affect the gut microbiome, the potential effects of PFAS on the skin microbiome may be of interest for further studies.

To summarise, there are indications in animal studies that exposure to certain PFAS can disrupt skin barrier integrity and modulate cytokine expression, potentially leading to inflammation and immune system dysregulation. While human epidemiological studies on PFAS-related skin effects are lacking, *in vitro* research provides insights into PFAS uptake and immunomodulatory effects on the skin. *In vitro* studies using human skin models have demonstrated PFAS uptake and inflammatory responses, although the concentrations used far exceed realistic exposure scenarios.

## Applicability of NAMs to study PFAS toxicity

Cell models of the lung, intestine and skin have already been used to investigate the hazards of several PFAS and their alternatives. There is no doubt that *in vitro* testing can, and should, be used for this purpose. However, there are several challenges in the use of *in vitro* studies that need to be addressed and considered, some of which will be discussed in the section below.

### Ensuring an accurate understanding of the effective concentration

*In vitro* to *in vivo* extrapolation (IVIVE) is an integral component of *in vitro*-based risk assessments. An accurate comprehension of the effective concentration in the cell can aid the extrapolation between *in vitro* and *in vivo* exposure and is as such of pivotal importance for an accurate risk assessment of PFAS. Yet, translating findings from *in vitro* studies to whole organisms remains a challenging task. This is in part due to the fact that *in vitro* systems are simplified models of the reality with the possibility that the effects observed at the cellular level may not necessarily reflect the responses of entire organs or systems in an intact organism. One reason for this may be differences between organ and tissue concentrations and those applied *in vitro*. However, *in vitro* studies in combination with state-of-the-art microscopy allow cellular localisation of halogenated compounds as well as to semi-quantitatively calculate concentrations of such compounds, which may to some extent enable *in vitro* to *in vivo* extrapolations (Gutleb et al. 2012).

#### Exposure concentration

The exposure/nominal concentration (*i.e.* the theoretical concentration of a test substance) used *in vitro* (e.g. 1–100 μM which, given that the molecular weight of PFOA is 404.07 g/mol, corresponds to ∼400–40,000 ng/mL for PFOA) are usually high compared to reported human serum concentrations (i.e. PFOA median blood concentration of 27 ng/mL, range 0.25–17,557 ng/mL), even compared to an elevated exposure scenario in adults living close to a chemical plant (Steenland et al. 2009). Yet, the use of such high exposure concentrations *in vitro* may be justified for several reasons. *In vitro* systems, by design, focus on short-term exposures and often require higher concentrations to elicit measurable effects within limited timeframes. Whereas the real-life exposure scenario to PFAS is spanning over several years, the intrinsic properties of *in vitro* methodologies make such a setup impossible. *In vitro* experiments are usually conducted over short durations and with endpoint measurements at a limited number of time points, resulting in approaches that struggle to quantify potential chronic, cumulative effects over extended durations (Macko et al. 2021). Given these limitations, higher concentrations are often used to reveal early molecular events or hazard signatures that could otherwise remain undetected in acute, short-term testing. Yet, the absence of overt cytotoxicity at high concentrations *in vitro* does not necessarily preclude more subtle effects, including those related to immunomodulation, which may require prolonged exposure to manifest.

Striving to facilitate chronic exposure scenarios of *in vitro* technologies would take away one of their major advantages, that is that they are fast, which allows rapid testing of a high number of chemicals. This scalability is crucial for timely risk management of chemicals. Hence, there is a need to extrapolate these short exposure durations to understand potential chronic effects. Extrapolation between different exposure times may be used to fill data gaps in regulatory toxicology using experimental animal data, for example from a 28-day study to a 90-day study by a factor of three (ECHA 2012). More recently, this has been explored also in the scope of *in vitro* data to characterise the toxicodynamics of a response also as a function of time (Macko et al. 2021). It is noted that comparisons between nominal *in vitro* concentrations and *in vivo* serum levels must be made cautiously, acknowledging the limitations and purpose of each model system. Nevertheless, the current chemical legislation in the EU (EC 2006) requires the identification of an effect to obtain a point of departure to extrapolate from, which for PFAS sometimes requires the use of concentrations that seem unreasonably high. Which effect to choose for such an extrapolation should be carefully considered as each toxicity pathway has its own toxicodynamic behaviour. Moreover, the biological impact of a perturbed pathway must be considered when determining the hazard linked to a chemical exposure, as some adverse effects may have a more severe implication than others, for example DNA damage/mutation versus an observed increased metabolic activity in the cells.

#### Bioavailability and cellular uptake in in vitro models

When studying PFAS effects using cell-based methods, factors influencing the bioavailability of the chemical to the cells should be considered. Specifically, the possible differences in the free fraction of PFAS *in vitro* versus *in vivo*, which has a relatively low free fraction due to the substantial serum binding (Xu et al. 2020; Zhang et al. 2020; Jackson et al. 2021). FBS in the culture medium has been shown to attenuate the cytotoxic activity of PFOS and PFOA (Giménez-Bastida et al. 2015; Zhang et al. 2020). However, an increased susceptibility to PFAS toxicity in an FBS-reduced culture may in part be attributed to the fact that the cells are more susceptible to chemical-induced damage due to a lack of important nutrients and hormones. Most of this effect, however, can probably be attributed to PFAS binding to albumin resulting in a lower fraction of chemical available to interact with the cells (Forsthuber et al. 2020; Jackson et al. 2021). PFAS have exceptionally low surface tension (Leung et al. 2023) and their amphipathic properties (i.e. having both hydrophilic and hydrophobic parts) cause them to stick to surfaces and interfaces. This means that in cell culture medium, PFAS may end up only at the water-air interface, much like an oil film on water. In theory, this can imply that adherent cells attached to the bottom of a culture vessel may not come into contact with the PFAS accumulating at the surface. To overcome this, PFAS-to-albumin binding can be utilised to shield their hydrophobic character, thereby facilitating PFAS delivery and uptake by the cells (Moro et al. 2022; Pye et al. 2023). This is somewhat contradictory to what has been seen in the literature, *i.e.* that FBS-deprived cells are more susceptible to PFAS exposure, and most likely this issue is more complex. Still, using a completely FBS-free cell culture medium without complexing the chemical to albumin through the addition of, for example bovine serum albumin may have a substantial impact on the cellular uptake of PFAS.

Albeit vital for accurate exposure assessment, as discussed above, there is limited reporting on the extent to which PFAS are taken up in the cells. The cellular internalisation of PFAS in cell cultures using 10% FBS has been demonstrated to be low. HepG2 cells exposed to a variety of PFAS (10, 30, and 100 μM for 24 h) internalised only 0.1%–4% of the dispensed concentration. While the intracellular concentrations increased with increasing treatment concentrations of the PFAS, the intracellular concentration expressed as a percentage of the administered amount of the tested PFAS remained fairly constant for the three treatment concentrations. When comparing the PFAS at non-cytotoxic concentrations (defined by the authors as >80% cell viability at tested concentrations) with the same carbon chain length, the cellular concentration was higher for PFCA than PFSA, which indicates that the functional group plays a role in the uptake of PFAS (Rosenmai et al. 2018). Also, the extracellular pH has an impact on uptake of PFOA. This was demonstrated using Caco-2 cells exposed to 1 μM PFOA in Hanks’ balanced salt solution without FBS and without complexing it to any other protein. Uptake of PFOA increased with decreases in extracellular pH (Kimura et al. 2017). Most cell lines function optimally around a pH of 7.0 to 7.4 and it is in this environment most toxicity assays are performed. The human intestine, however, varies in pH between 5.7 and 7.4. This could, in theory, lead to an underestimation of PFAS uptake in *in vitro* assays performed at a pH of 7.4. Cellular uptake has also been investigated in A549 cells exposed to 400 µM PFOS (which decreased the cell viability to roughly 60%), PFOA and GenX (non-cytotoxic concentration, ≥100% cell viability) with 10% FBS for 48hours. The intracellular concentrations of PFOA and PFOS were measured at 0.27 ± 0.35 µM/mg of cells and 18.2 ± 3.4 µM/mg of cells, respectively, whereas GenX was not detected. Still, GenX induced metabolic activity in the cells at the tested concentration (Jabeen et al. 2020). This contrasts with Rosenmai et al. (2018) where PFOA was taken up to a greater extent by the cells compared to PFOS. The difference in both exposure concentration and exposure duration between the studies may play a role but it cannot be excluded that the internalisation differs between cell types. Nevertheless, this highlights the importance of considering the protein content (i.e. FBS and/or albumin) in the cell culture medium and careful reporting of such to facilitate comparison between, and joint consideration of, *in vitro* studies of PFAS toxicity.

#### Intracellular localisation

The localisation of PFAS in individual cells is an important aspect that is rarely investigated, both *in vivo* and *in vitro*. Yet, the localisation of PFAS at tissue and sub-cellular levels could strengthen our understanding of their potential effects. It has been shown that the cellular concentrations of long-chain PFCA was higher than that of the shorter chains, while causing lower PPARα activity. The authors hypothesised that this was due to the higher cell membrane binding of long chain PFCA, rather than an actual uptake into the cell (Rosenmai et al. 2018). However, this hypothesis was not supported by imaging. There are methods available to quantitatively detect and determine the spatial distribution of some PFAS *in vivo* such as matrix-assisted laser desorption ionisation mass spectrometry imaging (MALDI MSI) (Li et al. 2021; Stoffels et al. 2023). However, the limit of the spatial resolution of such techniques does not allow localisation at the cellular level. Therefore, these detection methods are challenging to use with *in vitro* models. To quantify PFAS content *in vitro* other high-resolution techniques are required. Nanoscale secondary ion mass spectrometry (NanoSIMS) analysis has demonstrated the possibility to detect and localise PFAS in plants (Awad et al. 2022), in the humanadrenocortical cell line H295R (Gutleb et al. 2012), and in neurons (Berntsen et al. 2017). Recent analytical developments for nanoscale chemical imaging, such as focused ion beam (FIB)–scanning electron microscopy (SEM) coupled to secondary ion mass spectrometry (SIMS) (FIB-SEM-SIMS) instrument (De Castro et al. 2022), has also been applied to localise fluorinated compounds at the cellular level. With this instrument, PFOA was localised and quantified in the cytosol of intestinal cells (Stoffels et al. 2024). As such, identifying the subcellular localisation, ideally the direct quantification in the region of interest, of PFAS may contribute to elucidating the mechanism of action.

### Accounting for the susceptible window of (human) exposure to environmental pollutants in in vitro testing

A susceptible window of chemical exposure refers to specific periods during development or life stages when an organism may be particularly vulnerable to the adverse effects of chemical exposure. Certain biological pathways and processes may be more susceptible to disruption by chemicals. The time during which such critical pathways are under development or maturation (*e.g.* neurodevelopmental pathways, endocrine pathways, immune system development) represents susceptible windows during which exposure to chemicals can have profound and lasting effects on growth, differentiation, organogenesis, and functional maturation (Wright 2017). For PFOS and PFOA an association between maternal serum concentrations at 24 weeks of pregnancy to non-atopic asthma at age six years was found (Sevelsted et al. 2023). Recognising and considering these susceptible windows also in *in vitro* studies will be crucial to accurately assess the potential adverse effects of all chemicals, including PFAS. The undertaken approach must obviously be tailored to a specific hypothesis but may include one of the following: (i) cell culture models that mimic the developmental stages of interest, such as foetal or neonatal cells, (ii) exposure of cells at specific time points that correspond to the susceptible window, (iii) use of functional assays to evaluate specific developmental processes, such as neurodevelopment, cardiogenesis, or immune system maturation.

### PFAS mixtures

Although humans are exposed to a complex mixture of PFAS, most of the existing studies have focused on the toxicity of individual compounds, with only a few studies considering their mixture effects. It is not yet known if combined exposure to mixtures of PFAS – also known as the “cocktail effect” – may create interactions or synergistic effects among different PFAS chemicals. However, some studies have indicated that certain PFAS may exert a synergistic effect (Ojo et al. 2020; Pierozan et al. 2023). Understanding how these compounds interact and their combined impact on human health is a particularly complex task that needs to be addressed but will not be discussed further herein.

## Conclusion and outlook

The harmful effects of legacy PFAS compounds unfolded decades after substantial release into the environment and exposure to the human population began. The EU’s current regulatory perspective on PFAS demonstrates a proactive and science-based approach to safeguarding the environment and its’ citizens. Yet, PFAS pollution to the environment and human exposure to PFAS remain a major area of concern, while the list of known PFAS compounds continues to expand at a rapid pace. The large knowledge gaps regarding the human health effects of especially newer PFAS highlight the complexity of this issue. Based on the current knowledge, EFSA and the US EPA view decreased vaccine response as the most critical effect. In this paper, we set out to scrutinise the possible immune effects related to PFAA exposure at three biological barriers related to the major routes of exposure, that is the lungs, intestine, and skin.

Available research suggests that exposure to certain PFAS may exacerbate allergic asthma symptoms, alter airway function, and induce airway inflammation in animal models. Human studies present inconsistent associations between PFAS levels and lung function or asthma prevalence. Some studies demonstrate decreased lung function in children with asthma exposed to certain PFAS, while others show no significant associations or inconsistent results. Remarkably, recent studies suggest potential links between maternal PFAS concentrations and non-atopic asthma in offspring, warranting further investigation. Various *in vitro* studies have explored the immunomodulatory effects of PFAS, revealing, for some of the tested PFAS, a potential to alter cytokine release, inflammasome activation, and metabolic pathways in lung cells. Hence, while the literature offers insights into potential mechanisms linking PFAS exposure to immune toxicity outcomes in the lung, the findings remain fragmented. Increased serum levels of certain PFAS have been linked to the intestinal disease late-onset UC. However, evidence remains inconsistent, with some studies finding no clear associations between PFAS exposure and IBD risk. PFAS may affect the integrity of tight junctions in the intestinal epithelium, potentially increasing permeability and allowing environmental antigens to trigger immune responses associated with intestinal autoimmune diseases. *In vitro,* certain PFAS compromise tight-junction integrity and influence inflammatory responses in intestinal cells. However, findings are varied, with some studies indicating suppression of inflammation, contrary to expectations for IBD. Dermal uptake of PFAS into the systemic circulation in humans is not entirely clear. Yet, certain PFAS, including alternatives, have shown penetration and toxic effects on rodent skin. Rodent studies suggest potential disruption of the dermal barrier and immune toxicity through modulation of cytokine levels and gene expression. With some PFAS, *in vitro* studies using human skin models have shown uptake and inflammation induction, although with limitations in replicating real exposure scenarios.

It must be stressed that the majority of currently available human data on PFAS consists of epidemiological studies where blood concentrations of PFAS (mainly PFOA and PFOS) have been measured globally in various populations, both in the general population and in sub-populations exposed to PFAS in an occupational setting (EFSA CONTAM Panel 2020). Many of these studies are based on single measurements of PFAS serum concentrations, which neglects the exposure scenario and confounding factors, thereby limiting the interpretation of causality between PFAS exposure and the biological impact. Overall, while some epidemiological studies discussed in the present paper suggest possible immune-related effects of PFAS, the majority report no association or only weak and inconsistent findings, limiting the strength of conclusions that can currently be drawn. For the case of asthma, this uncertainty is further compounded by the heterogeneity of the disease, age-dependent expression, varying diagnostic criteria, and the influence of co-exposures and comorbidities (Peters 2014; Dharmage et al. 2019; Hudey et al. 2020), all of which make it particularly difficult to establish robust associations in epidemiological studies. Similar challenges likely apply to the evaluation of IBD, where diagnostic uncertainty and the complex, multifactorial aetiology of the disease complicate the interpretation of epidemiological associations (Loddo and Romano 2015; Xu et al. 2020). These issues reinforce the need for mechanistically informed studies.

Instead, a more mechanistic approach making use of NAMs to investigate chemical-induced adverse effects could be utilised. A mechanistic approach will aid understanding and quantification of the underlying biological and chemical mechanisms by which PFAS may exert toxic effects on organisms as well as the effect magnitude and concentration-response relationships. As such, this will provide a more detailed and predictive understanding of toxicity, beyond just empirical observations or correlations. This is, however, not without obstacles. Differences in experimental design, such as different concentrations, exposure duration, and cell/tissue systems, may explain the potentially contradicting results observed in many of the *in vitro* studies reviewed in the present paper. An important limitation of the available *in vitro* data is the lack of information on cellular uptake and the resulting internal concentration. Albeit such measurements could provide a better comparison with actual exposure levels, intracellular concentrations are rarely studied. Furthermore, as the experiment is not performed in an intact organism, there is a need to extrapolate the concentrations used to an appreciated *in vivo* dose. Extrapolating between test systems and accounting for differences in exposure durations is challenging but essential for understanding potential chronic effects. Therefore, the interpretation of *in vitro* findings must go beyond a simple comparison of nominal concentrations with human exposure levels. Higher concentrations are often necessary *in vitro* to trigger measurable effects within the short exposure windows characteristic of these systems. While the absence of cytotoxicity may confirm test system integrity, it should not be interpreted as a prerequisite for biological relevance. Ultimately, the critical issue is not the absolute concentration used, but whether the chosen test conditions yield results that are predictive of clinical relevance. Comparisons between *in vitro* concentrations and human biomonitoring data must be made cautiously, with full awareness of the methodological and biological context of each test system.

Central to a mechanistic approach is the identification and understanding of the MoA by which a chemical interacts with biological systems to produce adverse effects. This involves determining the specific molecular, cellular, or physiological processes that are perturbed by the chemical. By understanding the mechanistic basis of toxicity, it becomes feasible to extrapolate risks across different exposure scenarios or populations. This can aid the development of more robust risk assessments and regulatory decisions that account for variability and uncertainty. For PFAS, this requires further elucidation of the potential adverse effects associated with the exposure, which is no doubt a heavy burden. However, future risk assessment of PFAS is essential to protect human health, safeguard the environment, inform regulatory decision-making, and ensure the responsible management and use of these widespread substances.

## Glossary

CCL: Chemokine (C-C motif) ligand
CD: Crohn’s disease
CXCL: interferon gamma-induced protein
EpiDermFT: The EpiDerm Full Thickness
EU: European Union
FBS: foetal bovine serum
FEF25-75%: forced expiratory flow 25-75%
FEV1: forced expiratory volume in 1s
FIB: focused ion beam
FVC: forced vital capacity
GI: gastrointestinal
GM-CSF: granulocyte-macrophage colony-stimulating factor
IBD: inflammatory bowel disease
IgE: immunoglobulin E
IL: interleukin
IVIVE: *In vitro* to *in vivo* extrapolation
MALDI MSI: matrix-assisted laser desorption ionisation mass spectrometry imaging
MMP9: metalloproteinase 9
MoA: mode of action
MRP: multidrug resistance-related protein
NAMs: new approach methodologies
(Nano)SIMS: (nanoscale) secondary ion mass spectrometry
NASEM: National Academies of Sciences, Engineering, and Medicine
NLRP3: NOD-like receptor protein 3
OAT: organic anion transporter
OATP: organic anion-transporting polypeptide
OECD: The Organisation for Economic Cooperation and Development
OSTα/β: organic solute transporter-alpha/beta
PFAA: Perfluoroalkyl acids
PFCA: perfluorocarboxylic acids
PFSA: perfluorosulfonic acids
PFAS: Perfluoroalkyl and polyfluoroalkyl Substances, *NB PFAS is considered a plural noun.*
PFOA: perfluorooctanoic acid
PFOS: perfluorooctanesulfonic acid
Poly I:C: polyinosinic-polycytidylic acid
REACH: Registration, Evaluation, Authorisation and Restriction of Chemicals
SEM: scanning electron microscopy
TNF: tumour necrosis factor
UC: ulcerative colitis
US EPA: United States (of America) Environmental Protection Agency
